# Enhancers in T Cell development and malignant lesions

**DOI:** 10.1038/s41420-024-02160-7

**Published:** 2024-09-17

**Authors:** Tong Zhang, Lin Zou

**Affiliations:** 1 Clinical Medicine Research Department, Shanghai Children’s Hospital, Shanghai Jiao Tong University School of Medicine, Shanghai, 200062 China; 2 Postgraduate School in Shanghai Jiao Tong University School of Medicine, Shanghai, 200025 China; 3 Institute of Pediatric Infection, Immunity, and Critical Care Medicine, Shanghai Children’s Hospital, Shanghai Jiao Tong University School of Medicine, Shanghai, 200062 China

**Keywords:** Acute lymphocytic leukaemia, Lymphoma

## Abstract

Enhancers constitute a vital category of cis-regulatory elements with a Mediator complex within DNA sequences, orchestrating gene expression by activating promoters. In the development of T cells, some enhancers regulate the critical genes, which might also regulate T cell malignant lesions. This review is to comprehensively elucidate the contributions of enhancers in both normal T cell development and its malignant pathogenesis, proposing the idea that the precise subunits of the Mediator complex are the potential drug target for disrupting the specific gene enhancer for T cell malignant diseases.

## Facts

- The focus points about enhancer roles in human T cell development and differentiation were concentrated on TCR, CDs, and TFs.
- The concept of super-enhancer distinguished the characteristics of different enhancers and provided us with a new perspective on enhancer-driven pathogenic genes.
- Disruption of the enhancers, including gene mutation became one of the most important causes of T cell malignancies.
- The existing enhancer function inhibitors including BET or CDK inhibitors have achieved certain therapeutic effects in clinical trials, but at the same time, also brought numerous side effects because of they generally lacking specificity.
- The human Mediator complex is expected to serve as a specific target for blocking the related enhancer-promoter looping structure, especially the MED1 and MED12 subunits, which can bind to specific eRNAs.

## Open questions

- Can we find a better way to dynamically monitor the role of enhancers in the T cell differentiation process?
- Is it necessary to establish one enhancer database, whose contents include the unified standard enhancer ID, the enhancer sequences, the enhancer-driven genes, super-enhancer identification, etc.?
- Do the human T cell lymphotropic virus have their preferences when they infect human T cells and select insertion DNA sequence sites?
- How to develop targeted drugs that can bind specifically to different eRNAs and disrupt related enhancer–promoter looping structures?

## Introduction

T lymphocytes originate from early lymphoid progenitors (ELPs), which develop lymphoid-primed multipotent progenitors [LMPPs]/common lymphoid progenitors [CLPs]), migrate from the bone marrow (in adults) or liver (during fetal life) to the thymus, where they undergo a series of developmental processes including differentiation, selection, maturation, and eventual export to peripheral tissues. The thymus provides a specialized microenvironment conducive to T lymphocyte development and continuously receives input from ELPs, which acquire a definitive T cell identity to generate mature αβTCR and γδTCR T cells. Early T cell precursors (ETPs), the first phenotypically distinct T cell progenitors derived from LMPPs/CLPs, exhibit a specific cell surface marker phenotype (CD3^−^/CD4^−^/CD8^low^/CD25^−^/CD44^hi^/KIT^+^) [1]. Under the influence of cytokines and transcription factors, ETPs undergo expansion and differentiation into CD4^−^CD8^−^ double-negative (DN) T cells, which subsequently express TCR. DN T cells undergo TCR rearrangement to generate CD4^+^CD8^+^ double-positive (DP) T cells. DP T cells undergo negative selection at the corticomedullary junction of the thymus, giving rise to CD4^+^ or CD8^+^ single-positive (SP) T cells that ultimately enter the periphery as naive T cells exhibiting CD45RA^+^CCR7^+^ phenotypes [2, 3]. In these stages of T cell development, the regulation of critical genes contributes to its normal function.

Throughout the normal functions of T cell development related to the genomic DNA, including gene enhancer sequences, undergoes accurately orchestrated regulation by a complex network of signaling pathways, ensuring the proper execution of each developmental stage. Effective T cell development, maturation, and functions in vivo hinge upon the accurate progression of each phase, which enhances their important role. Deviations from the correct developmental trajectory, often accompanied by genetic mutations, can result in cell cycle arrest and the onset of malignant diseases. Each stage of T cell development manifests distinct characteristics and relies on the precise orchestration of specific regulatory factors. This review mainly summarizes the pivotal roles of enhancers within the intricate regulatory landscape of T cells, the T cell malignancies, as well as the related clinical trials based on enhancers for T cell leukemia/lymphoma. Notably, we provide the idea that targeted intervention for specific gene enhancers may be a potential therapeutic approach for T cell malignancies.

## Characteristics and identification of enhancers

Since the establishment of DNA as the genetic material and the discovery of its double-helix structure in the last century, persistent efforts have been directed towards unraveling the functions of each DNA fragment [4]. Increasingly, the traditional non-coding regions of DNA are recognized to play crucial roles in the development and differentiation of cells. The concept of enhancers was initially proposed in 1981 when a 72 bp repeated DNA segment was identified to be capable of enhancing SV40 globin gene expression [5]. Although enhancers exhibit a close functional relationship with promoters, they diverge significantly from them. Enhancers are typically considered to target promoters and augment their abilities but not target genes directly. Consequently, enhancers increase the expression of target genes through enhancer-promoter communication. Various components engage in the active enhancer–promoter looping structure, including Mediator complex, bromodomain-containing protein (BRD), cyclin-dependent kinases (CDKs), RNA polymeraseII (RNA-PolII), transcription factors (TFs), and methylated promoter [6].

The enhancer, as an inherent element of DNA, drives naturally the related-gene expression. Certainly, the enhancer functions are influenced by chromatin accessibility, histone modification, DNA mutation, TFs recruitment, and cytokines exposure. These interfering conditions have a more significant impact on super-enhancer functions. Briefly, the higher levels of chromatin accessibility, H3 lysine 27 acetylation (H3K27ac), and histone H3 lysine 4 monomethylation (H3K4me1), together with the lower level of trimethylation of histone H3 lysine 4 (H3K4me3) are indeed a representative hallmark that enhancers are more active. TFs recruitment is a necessary step before enhancer plating its roles. DNA mutation, including chromatin structural variations (SVs), could revolutionize the enhancer functions, over activation or complete silence. Some cytokines, such as prolactin and interleukin-2, strengthen the functions of some specific enhancers [7].

In recent decades, novel methodologies have been established to assess potential sequence sites of enhancers. Particularly, H3K27ac is a prominent hallmark of gene enhancers. Abundant new enhancers using H3K27ac reporter assays have been identified [8]. Additionally, H3K4me1, another evolutionarily conserved feature of enhancer chromatin, is enriched at active and primed enhancers while being depleted in H3K4me3 [9, 10].

However, recent insights gained through CRISPR/Cas9 methods coupled with single-cell RNA sequencing unveil that only approximately 10% of enhancers identified by H3K27ac characteristics demonstrate actual enhancer activities. Furthermore, the development of massively parallel reporter assays (MPRA) seeks to elucidate the precise spatial and temporal activity of enhancers, promising extensive application across diverse specimens [11]. Regarding H3K4me1, it remains ambiguous whether it governs or merely correlates with enhancer activity and function [12].

Therefore, recent studies aim to identify genome enhancers that typically incorporate two or more enhancer characteristics. For instance, researchers may consider both high levels of H3K27ac and H3K4me1, or high levels of H3K27ac and H3K4me1 combined with low levels of H3K4me3. In an effort to better discriminate enhancers with varying activities and their capacity to regulate gene expression levels, Professor Young first introduced the concept of super-enhancers in 2013 as a departure from conventional enhancers, which must meet four criteria: longer sequence size, higher transcription factor density and content, stronger activate transcription ability, and greater sensitivity to perturbation, thus distinguishing them from typical enhancers [13, 14].

However, the quantitative standards to evaluate whether an enhancer qualifies as a super-enhancer are still lacking. In subsequent reports on super-enhancers, researchers divided super-enhancers according to their own understanding criterion. Nevertheless, the concept of super-enhancers carries significant implications for the field of enhancer research.

## The roles of enhancers in normal t cell differentiation and development

The differentiation and development of normal T cells encompass several crucial stages, commencing with the migration of hematopoietic stem cells (HSCs) into the thymus. These HSCs undergo progressive differentiation into ETP, CD4^−^CD8^−^ DN T cells, CD4^+^CD8^+^ DP T cells, and ultimately CD4^+^CD8^−^/CD4^−^CD8^+^ SP T cells, following a predetermined sequence [2].

Accumulating studies have provided valuable insights into the roles of enhancers in normal T-cell differentiation and development. Particularly, the investigation into enhancers’ regulation of T cell differentiation and development garnered significant interest during two distinct periods: around 2000 and after 2015. The latter flow in interest can be attributed to the revolutionary advancements in detection technologies and experimental methods related to enhancers. For instance, in 2007, H3K4me1 was first utilized to predict enhancer chromatin, followed by the recognition of H3K27ac as a widely accepted identification standard for active enhancers in 2010 [15]. Furthermore, the advent of CRISPR/Cas9 technology facilitated the analysis of human genome enhancers, starting in 2016 [16].

In this part, we summarize the related enhancers and their functions across several critical stages of T cell differentiation and development (Table 1).

**Table 1:** Overview of enhancer roles in normal T cell development.

T cell stages	Enhancers	Relevant genes	Functions	Research model	Reference
ETP	E^BAB^, GATA2/NOTCH1 enhancer	Bim, GATA2, NOTCH1	TCR activation and differentiation	Targeting gene knockdown mice	[17–19]
DN	Eα, Eδ, Eβ, E8I-V, pTα/Pu.1/HES1/HES5/CD4/ETS1 enhancer	pTα, E47, Pu.1(Sfpi1), Pip-1, RUNX1, NOTCH1, BEAD1, CD4, E2A, HEB, NOTCH3, HES1, HES5, CD8, HOXA5-9, ETS1, RUNX3,	TCR differentiation, CD4/CD8 activation, NOTCH signal pathway, HOXA5-9 transcription	Targeting gene knockdown mice, T cell lines	[22–27, 29, 30, 34–39, 75]
DP	Eα, Eδ, Eβ, E8I-V, CD4/FOXP3/ZAP70 enhancer	CD4, FOXP3, CD8, ZAP70, Mi2b, HEB, E2A, p300	CD4/CD8 activation, TCR differentiation and rearrangement	Children’s thymic tissue, targeting gene knockdown mice, T cell lines	[21, 24, 31, 33, 42–47, 75]
SP	Eα, Eδ, E8I-V, E4p, E4m, CD4/FOXP3 enhancer	CD4, FOXP3, CD8, LEF1, TCF1, ZEB, TET1, TET3	CD4/CD8 selected expression, T cell phenotype differentiation	Targeting gene knockdown mice, T cell lines	[20, 28, 32, 40, 41, 76]

*E*^*BAB*^ enhancer of Bub1-Acoxl-Bim, *GATA2* guanine–adenine–thymine–adenine binding protein 2, *Eα* TCR alpha enhancer, *Eδ* TCR δ enhancer, *Eβ* TCR β enhancer, *pTα* pre-TCR alpha, *E47* basic helix–loop–helix transcription factor E47, *Pu.1* Ets family transcription factor Pu.1, *Pip1* Pu.1-interacting protein, *Sfpi1* spleen focus forming virus proviral integration 1, *CSL* CBF1/RBP-Jkappa/Suppressor of Hairless/LAG-1, *BEAD1* blocking element alpha/delta 1, *E2A* basic helix–loop–helix transcription factor E2A, *HEB* HeLa E-box binding protein, *HES1/5* Split homolog 1/5, *E8I-V* CD8 enhancer I–V, *LEF1* lymphoid enhancer-binding factor 1, *TCF1* T cell factor 1, *FOXP3* forkhead box P3, *ZAP70* zeta-chain associated protein kinase 70, *Mi2b* chromatin remodeling factors Mi2b, *p300* histone acetyl transferase p300, *ZEB* zinc-finger E-box-binding transcriptional repressor, *E4p* CD4 proximal enhancer, *E4m* CD4 “maturity” enhancer, *TET1/3* ten–eleven translocation family 1/3.

## Etp

Research on the roles of enhancers in ETP is relatively limited. Bim is reported to be genetically required as a downstream target of TCR signaling for establishing central T cell tolerance and depleting activated T cells in the periphery. The T cell-specific genomic enhancer E^BAB^ regulates the Bcl-2 Interacting Mediator of cell death (Bim) gene expression in ETP, thereby contributing to the promotion of thymic negative selection and the suppression of autoimmunity [17]. Additionally, the absence of the GATA binding protein 2 (GATA2) enhancer and/or notch receptor 1 (NOTCH1) enhancer leads to a reduction in the number of ETP, accompanied by accelerated T cell apoptosis and aberrant differentiation [18, 19].

### DN and DP T Cells

DN and DP stages represent critical phases of normal T cell differentiation and development throughout the entire T cell lifecycle. The question of how enhancers influence the transition from DN to DP stage T cells has been a focal point since the introduction of the concept of enhancers. Early research predominantly concentrated on identifying enhancers related to T lymphocytes [20, 21]. Enhancers play vital roles in TCR expression and rearrangement, encompassing almost every part of the TCR, including TCR α, β, γ, and δ [22–28]. Not only do enhancers of the TCR contribute to this process, but enhancers of related genes also regulate normal differentiation and development processes from DN to DP stages. For instance, enhancers of HES1/5 have been implicated in this transition [29]. Additionally, CD4 and CD8, characteristic positive markers of T lymphocytes, are regulated by enhancers controlling their expression from DN to DP stages [30].

TFs, for example, LEF1 [31, 32], TCF1 [33], E47 [34], NOTCH1 [23], RUNX1 [35], and E2A-HEB [25], bind to TCR enhancers, initiating TCR expression. Conversely, BEAD1 serves as an enhancer-blocking element, preventing the TCR δ enhancer from activating TCR δ gene segment transcription and rearrangement [36, 37]. The PU.1 enhancer exhibits myeloid-specific activity, activating the PU.1 gene in myeloid cells while remaining silent in lymphocytes [38]. ETS1 enhancer activity is attained at the DP stage, facilitating the transition of DN to DP thymocytes [39]. However, E47, in combination with PU.1, exerts a synergistic effect on the activity of the pTα enhancer in DN stage T cells [34].

### SP T cells

The SP stage marks the traditional formation of mature lymphocytes. In CD4^+^CD8^−^ SP stage T cells (T helper), the CD4 gene is regulated by three distinct enhancers (Eα/β/δ) for its expression [20, 21]. Furthermore, enhancers of related genes exert a significant influence on CD4 gene expression. For instance, E4p, in combination with E4m, promotes CD4 gene expression by facilitating CD4 gene demethylation [40]. On the contrary, zinc finger/homeodomain transcription factor (ZEB) acts to silence the CD4 proximal enhancer [41]. Upon transition from the DP stage to the CD4^+^CD8^−^ SP stage, innumerable factors, including ZAP70 [42], Mi2b, HEB, E2A, and p300 [43], become inactive.

In addition, demethylated forkhead box protein 3 (FOXP3) is associated with a stable Treg phenotype [44]. In CD4^−^CD8^+^ SP stage T cells (T cytotoxic) [26, 45–47], there are six diverse CD8 enhancers (E8I-VI) regulating CD8 gene expression. All E8I-VI enhancers are required for CD8 expression in DP and CD4^−^CD8^+^ SP stage T cells.

## The regulated functions of enhancers in t cell malignancies

The profound effects of enhancers on the occurrence and progression of T cell malignancies manifest in two main aspects: T cell genomic enhancement abnormally changing (Table 2), and leukemia virus enhancers inserting T cell DNA sequences (Table 3). It is worth noting that certain fragments of leukemia virus DNA can interfere with T-cell gene enhancers. These mutations disrupt the normal expression of wild-type T-cell DNA, consequently heightening leukemogenicity.

**Table 2:** Overview of enhancer roles on T cell leukemia/lymphoma.

Disease	Enhancer	Related genes	Mutant site	Functions	Research model	Reference
T-ALL	Eα, Eδ, Eβ, GIMAP/MYC/TP73 enhancer	GIMAP, TAL1, NOTCH1, TCF1, MYC, IL2RA(CD25), CD30, FYN, TIAM2, TP73	TAL1, E-proteins, MYC, TP73 exons 2–3	TCR translocation, impairing T-ALL development, T cell activation pathway,	Patients’ primary leukemia cells, targeting gene transgenic zebrafish, T-ALL cell lines	[49, 50, 54–56]
Lymphoma	Eβ, BCL11b/TOX2 enhancer	PDCD1, CXCR5, BCL6, BCL11b, BATF3, IL2R, TOX2	Eβ	aberrant V(D)J cleavages, blocking T cell development and developing lymphoid malignancies	Primary human Tfh and Teff cells, targeting gene transgenic mice, lymphoma cell lines	[48, 77–80]

*T-ALL* T cell acute lymphoblastic leukemia, *Eα* TCR alpha enhancer, *Eδ* TCR δ enhancer, *Eβ* TCR β enhancer, *GIMAP* GTPase of immunity-associated protein, *TAL1* T cell acute lymphoblastic leukemia 1, *IL2RA* interleukin 2 receptor alpha, *TIAM2* T cell lymphoma invasion and metastasis 2, *Tfh cells* T follicular helper cells, *Teff* T effector cells, *BATF3* basic leucine zipper atf-like transcription factor 3, *IL2R* interleukin 2 receptor.

**Table 3:** Overview of virus enhancer roles on T cell leukemia/lymphoma.

Disease	Virus	Enhancer	Related genes	Mutant site	Functions	Research model	Reference
T-ALL	HTLV1, M-MuLV	HTLV1 PuB2/p30II/Tax, HTLV1/MuLVs enhancer	ETS1, ELF1, MYC, EPC1, SRF, ELK1	PuB2, p30II, 10p11.2	Disturbing T cell cycle	Human/mouse T cell lines	[58, 59, 81–84]
Lymphoma	M-MuLV, SL3-3	MuLV/SL3-3/SV40 enhancer	RUNX1	Runx1	Inducing lymphomas	Mice, human/mouse/rat T cell lines	[60, 85–93]

*M-MuLV* moloney murine leukemia virus, *EPC1* enhancer of polycomb 1.

Research on virus DNA enhancers was relatively concentrated in the 1990s, as the shorter virus DNA sequences facilitated the elucidation of virus pathogenic mechanisms for T cells. Conversely, in the more complex T cell genome, research on enhancers is still in its initial stage, with the content being more extensive.

### Enhancers in T cell leukemia/lymphoma

Disruption of these enhancer elements can hinder T cell differentiation and development, ultimately leading to the onset of T cell leukemia/lymphoma. The TCR serves as a prime example. In normal T cells, the regulatory mechanism of TCR enhancers is relatively well-understood. However, aberrant functions of TCR enhancers can lead to TCR differentiation arrest and rearrangement disorders [48], often accompanied by chromosomal translocations [49].

The NOTCH pathway and its associated proteins regulated by gene enhancers, including MYC and TCF1, are significant pathogenic factors in leukemia. The TCF1-dependent NOTCH1-regulated MYC enhancer plays a fundamental role in shaping the leukemia-prone epigenetic landscape during the transition from preleukemic cells to full-blown disease [50].

Of particular, the enhancer of zeste homolog 2 (EZH2), though EZH2 is not a gene enhancer in humans, it shares a homologous DNA sequence with the enhancer of zeste in drosophilae. EZH2, as an epigenetic regulator, has a critical influence on global H3K27 methylation [51]. Overexpression of EZH2 is commonly observed in T lymphomas and linked to the pSTAT3- MYC pathway [52]. Patients with high EZH2 protein transcripts often exhibit a worse prognosis [53]. Moreover, some studies demonstrate that enhancers can give rise to transcriptional eRNAs (enhancer RNA), which target enhancer-related promoters and exist only briefly. EZH2 provides a compelling example that demonstrates how some enhancers’ sequences can transfer coding-gene functions in the biological evolution process, thereby playing more significant roles.

Furthermore, the driving effects of super-enhancers on oncogenes are garnering more attention, revolutionizing the traditional understanding of abnormal regulatory genes in T cells, to redefine the type, scope, and regulation methods of target genes. Existing evidence suggests that super-enhancers exhibit functional abnormalities in regulating HSC signaling, mitochondrial energy supply, and the expression of anti-tumor proteins in leukemia cells.

Through genomic-level analysis of super-enhancer profiling, researchers have identified multiple previously unreported super-enhancers that are abnormally active in leukemia cells. This discovery provides a new research avenue for further investigating abnormal lesions in leukemia cells. A newly characterized gene, T cell lymphoma invasion and metastasis 2 (TIAM2), is associated with super-enhancers in adult T cell leukemia/lymphoma (ALT) samples, but not in normal T cells [54]. Super-enhancers, including GIMAP, TP73, BCL11b, and MYC enhancers, contribute to inducing leukemia/lymphoma by regulating the expression of TF and cytokines [55, 56].

Importantly, abnormal regulation of gene enhancers can be accompanied by varying degrees of mutations at gene sites, eg. chromatin SVs, which may serve as important targets for drug intervention [57].

### Virus enhancers in T cell leukemia/lymphoma

In cases of leukemia/lymphoma induced by viruses such as human T cell lymphotropic virus type 1 (HTLV1), enhancer elements within the viral DNA sequence integrate into the T cell genome, resulting in abnormal expression of certain key proteins in T cells [58]. Currently, it is not fully understood whether leukemia viruses exhibit selectivity for some specific T cell genes that are interfered with by the viruses. Studies on HTLV1, the most common human leukemia virus, and murine leukemia virus (MuLV), commonly used in constructing mouse models in experiments, have shown that genes related to T cell development, in particular, ETS1, ELF1, SRF, and MYC, display functional abnormalities driven by viral enhancers upon T cell infection by the virus [59, 60].

The specific fusion genes driven by the virus enhancer, which plays a decisive role in the onset of leukemia/lymphoma, are still under investigation. A recent theory called enhancer hijacking-mediated oncogenic transcription, based on chromatin SVs, may shed light on the mechanism by which viral enhancers regulate T cell genes, suggesting that TAL1 could hijack the MYCN enhancer, leading to MYCN overexpression [61].

## Therapeutic strategies for interrupting abnormal enhancer activities in t cell diseases

The most direct approach to block enhancer-driven gene expression involves gene editing, like knocking out corresponding abnormal enhancer sequences. With the maturation of CRISPR technology, gene editing therapy holds promise as a future treatment method for correcting enhancer abnormalities. However, due to significant differences between in vivo human body environments and cultured cells in vitro, and incomplete understanding of the side effects caused by gene editing, these treatment methods remain confined to cultured cells in vitro or animal experiments.

The enhancer drives the promoter’s function through the enhancer-promoter looping structure. Thus, disrupting or blocking enhancer-promoter looping can effectively inhibit the enhancer’s function. However, the components involved in enhancer-promoter looping are complex, with numerous potential targets for interference.

In the regulatory mechanism of enhancer-promoter looping, the bromodomain and extraterminal (BET) protein family plays a pivotal role in the Mediator complex of RNA-PolII transcription, while CDK regulates the phosphorylation level of RNA-PolII. Consequently, BET or CDK inhibitors, primarily small molecules, represent broad approaches to restrain enhancers’ activities. Additionally, histone acetylation is a prerequisite for activated enhancers’ functions. Therefore, histone acetyltransferase (HAT) inhibitors can disrupt enhancers’ functions [62]. Correspondingly, modulating histone deacetylase (HDAC) expression levels also significantly influences enhancers’ functions.

However, there are limitations to using small molecular inhibitors to suppress enhancers’ activities. BET or CDK inhibitors act as pan-inhibitors, affecting all enhancer activities, such as those necessary for normal gene functions. A part of BET or CDK inhibitors are used as clinical drugs, but most of them lack specificity, which leads to adverse events (AEs) and off-target effects.

### Pre-clinical therapeutic strategies

Some BET or CDK inhibitors have been applied to treat T cell malignancies by disrupting abnormal enhancers’ activities. We have compiled published pre-clinical researches on targeting enhancer sites until 2024, summarizing their characteristics and treatment effects (Table 4).

**Table 4:** Summary of pre-clinical therapy based on enhancers in T-ALL.

Drug-name	Drug-target	Therapeutic effects	Research model	Reference
JQ1	The inhibitor of BET protein	Inducing T-ALL cell apoptosis, inhibiting proliferation	T-ALL cell lines, primary patient T-ALL cells	[94]
dBET6	The degrader of BET protein	Collapsing the core transcriptional circuitry of T-ALL	T-ALL cell lines, naive CD4^+^/CD45RA^+^ T cells, primary PDX samples	[63]
JQ1	The inhibitor of the BET protein	Reducing T-ALL cell viability, suppressing PDX cell growth	T-ALL cell lines, PDX	[95]
OTX015	The inhibitor of the BET protein	Suppressing T-ALL cell proliferation and promoting apoptosis, reducing tumor growth	T-ALL cell lines, PDX	[64]
ARV‑825	The degrader of BET protein	Inhibiting T-ALL cell proliferation, reducing tumor growth	T-ALL cell lines, PDX	[65]
JQ1, GSK343, OTX015	The inhibitor of BET protein and EZH	Prolong T-ALL PDX mice survival time	T-ALL PDX	[96]
I-BET151	The pan-BET inhibitor	Arresting cellular growth and proliferation	T-ALL cell lines	[66]
dinaciclib	The CDK inhibitor	Inhibiting T-ALL cell viability, inducing apoptosis, reducing colony formation capacity, and prolonging mice survival time	T-ALL cell lines, the mouse xenograft model	[67]
alvocidib	The CDK9 inhibitor	Inhibiting the growth of ATL cells both in vitro and vivo and inducing apoptosis and cell cycle arrest	ATL cell lines, primary ATL patients’ cells, and normal CD4^+^ lymphocytes	[69]
PIK-75	The inhibitor of PI3K and CDK	Inhibiting T-ALL cell growth and inducing Apoptosis	T-ALL cell lines, primary patient T-ALL cells	[68]

*PDX* patient-derived xenograft.

JQ1 is a typical and commonly used BET inhibitor. It has been applied alone or in combination with other BET inhibitors, for instance, GSK343 and OTX015, to suppress the proliferation of T cell acute lymphoblastic leukemia (T-ALL) cells, and promote their apoptosis. Furthermore, these treatments have shown significant improvements in the survival time of primary patient-derived xenograft (PDX) mice. Novel BET inhibitors continue to be discovered, including dBET6, OTX015, ARV-825, and I-BET151. dBET6 is particularly effective in degrading BET proteins, especially bromodomain-containing protein 4 (BRD4), in T-ALL cells. It disrupts the interaction between enhancers and promoters, leading to specific downregulation of phosphorylated RNA-PolII on Ser2, thereby disrupting the core transcriptional circuitry and global productive transcription elongation [63]. OTX015 specifically inhibits BRD2 [64], while ARV-825 is a highly effective BET protein degrader targeting BRD2, BRD3, and BRD4. ARV-825 exhibits a stronger anti-proliferative effect and lower IC50 than JQ1, dBET1, and OTX015 in T-ALL cell lines [65]. I-BET151, a pan-BET inhibitor, disrupts RUNX1-driven pathogenic super-enhancers by inducing a broad reduction in H3K27ac levels in T-ALL [66].

While fewer studies have focused on CDK inhibitors for T-ALL pre-clinical treatments compared to BET inhibitors, The CDK inhibitor dinaciclib suppresses c-MYC and cyclin T1, induces G2/M phase cell cycle arrest, and triggers apoptosis in T-ALL cells [67]. PIK-75, an inhibitor of PI3K and CDK, has been shown to reduce enhancer activities driven by TAL1 [68]. Additionally, Alvocidib specifically inhibits CDK9, reducing IRF4 expression via super-enhancer suppression [69]. These findings suggest the efficacy of BET and CDK inhibitors in treating T-cell malignancies by suppressing abnormal enhancer activity.

Currently, pre-clinical studies primarily focus on understanding the functions of enhancer inhibitors for T lymphocytes. These studies involve existing cell lines, primary T cells obtained from patients, and PDX mice models. The consensus among researchers is that disrupting aberrant high-active enhancers, some of which may be caused by gene structure variations, is necessary for effective treatment.

A new regulatory mechanism has emerged, highlighting the importance of histone acetylation and deacetylation processes mediated by HATs and HDACs, respectively. Interestingly, despite having opposite catalytic functions, both HAT inhibitors and HDAC inhibitors demonstrate anti-cancer effects [70, 71]. In the context of T-ALL research, more exploration has been conducted on the regulatory roles of HDAC inhibitors on enhancers [72].

Although most enhancer inhibitors have demonstrated superior cytotoxicity by promoting apoptosis and arresting the cell cycle in T leukemia/lymphoma cells, translating these findings into effective clinical therapeutic regimens remains challenging. There is still a long road ahead in terms of refining these inhibitors and developing clinical strategies that leverage their therapeutic potential effectively. Further research is needed to optimize their efficacy, minimize off-target effects, and establish safe and effective dosing regimens before they can be widely adopted in clinical practice. Additionally, the complexity of T leukemia/lymphoma and the heterogeneity among patients may necessitate personalized treatment approaches tailored to individual molecular profiles.

### Clinical trials completed or in progress based on enhancers

We list all representative clinical trials on clinicaltrials.gov, which investigate T cell leukemia/lymphoma interventional strategies with BET/CDK inhibitors treatment, based on enhancers (Table 5). Most of them are in study phase 1 and could be observed with antitumor activities. However, a majority of BET/CDK inhibitors in completed status clinical trials have been reported adverse events (AEs), for example, anemia, neutropenic fever, nausea, fatigue, thrombocytopenia, etc [73, 74]. Generally speaking, clinical therapeutic strategies of suppressing enhancer activities based on BET/CDK inhibitors partly achieve the goals, which still have a distance to be adopted as the formal clinical medication.

**Table 5:** Summary of clinical trial on the BET/CDK inhibitors based on enhancer in T cell malignancies.

Disease	Study ID	Drug-name	Synonyms	Drug-Target	Patients-number	Phase	Status
ALL	NCT03740334	Ribociclib	—	CDK inhibitor	45	I	Active, not recruiting
	NCT01701375	Palbociclib	PD-0332991	CDK4/6 inhibitor	2	I	Terminated
ALL/lymphomas	NCT03792256	Palbociclib	PD-0332991	CDK4/6 inhibitor	15	I	Active, not recruiting
CLL	NCT03739554	Fadraciclib	CYC065	CDK2/9 inhibitor	5	I	Completed
	NCT05168904	Fadraciclib	CYC065	CDK2/9 inhibitor	210	I & II	Suspended
	NCT01580228	Dinaciclib	SCH 727965	CDK1/2/5/9 inhibitor	44	III	Completed
	NCT01627054	AT7519M	—	CDK inhibitor	7	II	Completed
	NCT00446342	SNS-032	—	CDK inhibitor	21	I	Completed
CLL/lymphomas	NCT00871663	Dinaciclib	SCH 727965	CDK1/2/5/9 inhibitor	123	I	Completed
	NCT01515176	Dinaciclib	SCH 727965	CDK1/2/5/9 inhibitor	36	I & II	Completed
	NCT05665530	PRT2527	—	CDK9 inhibitor	104	I	Recruiting
	NCT03547115	Voruciclib	—	CDK9 inhibitor	100	I	Recruiting
NHL	NCT02543879	FT-1101	CC-95775	BET inhibitor	94	I	Completed
	NCT04089527	FT-1101	CC-95775	BET inhibitor	24	I	Completed
	NCT03220347	BMS-986378	CC-90010	BET inhibitor	139	I	Active, not recruiting
	NCT00871910	Dinaciclib	SCH 727965	CDK1/2/5/9 inhibitor	81	I	Completed
	NCT05758610	Euthare-155008	—	CDK4/6 inhibitor	60	I	Recruiting
	NCT00147485	AG-024322	—	CDK1/2/4 inhibitor	37	I	Terminated
	NCT00141297	Palbociclib	PD-0332991	CDK4/6 inhibitor	74	I	Completed
Lymphomas	NCT02711137	INCB057643	—	BET inhibitor	137	I & II	Terminated
	NCT02431260	INCB054329	—	BET inhibitor	69	I & II	Terminated
	NCT01943851	Molibresib	GSK525762	BET inhibitor	111	II	Completed
	NCT01949883	Pelabresib	CPI-0610	BET inhibitor	64	I	Completed
	NCT03936465	BMS-986158	—	BET inhibitor	41	I	Active, not recruiting
	NCT03936465	BMS-986378	CC-90010	BET inhibitor	41	I	Active, not recruiting
	NCT05053971	ZEN-3694	—	BET inhibitor	30	I & II	Recruiting
	NCT03925428	Molibresib besylate	GSK525762C	BET inhibitor	0	I	Withdrawn
	NCT04983810	Fadraciclib	CYC065	CDK2/9 inhibitor	330	I & II	Recruiting
	NCT01564251	GDC-0575	—	CDK inhibitor	104	I	Completed

*ID* identify, *NHL* non-Hodgkin lymphoma.

## Conclusion and perspectives

**Fig. 1:**
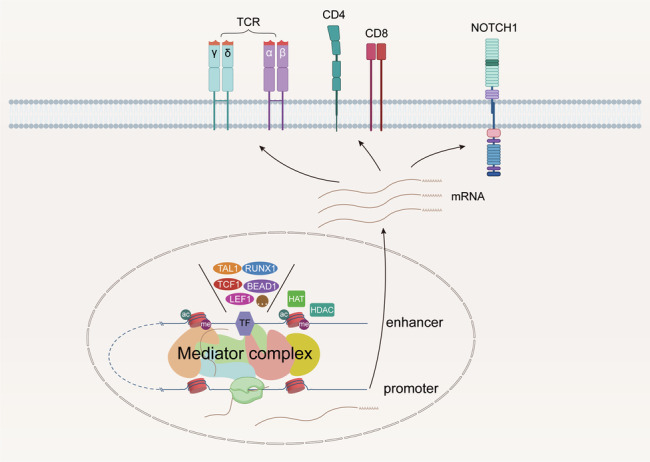
Regulation of the key genes in T cell development through enhancer-promoter looping. The enhancer recruits TFs, such as TAL1, RUNX1, TCF1, BEAD1, and LEF1, and enhancer–promoter looping becomes active. The enhancer drives the related promoter, then gene transcription starts. Current researches reveal that expression of TCRs, selection of CD4 or/and CD8, and NOTCH signal pathway in T cells are closely dependent on enhancers. Abbreviations: TF transcription factor, TCR T cell receptor, HAT histone acetyltransferase, HDAC histone deacetylase.

The essential roles of enhancers in T cell development are summarized (Fig. 1). Although the comprehension of the functions and roles of enhancers in T cell development and differentiation is limited, we notice that TFs binding enhancers regulate downstream genes, the most involved TCRs, CDs, NOTCH signaling pathway. Therefore, TFs have a pivotal influence on the process of enhancers driving promoters. More researches are needed to reveal the alternation of different enhancer active levels in different stages of T cells, which favors us with a better understanding of T cells tumorigenesis.

The concept of super-enhancers brightens us to establish a reasonably and systemically classificatory rules of enhancers. However, there is still no quantitative standard to identify whether an enhancer is typical-enhancer or super-enhancer. H3K27ac and H3K4me1 are generally adopted in evaluating enhancers, which supplies the possibilities of setting up one enhancer database for recording characteristics and functions of enhancers.

**Fig. 2:**
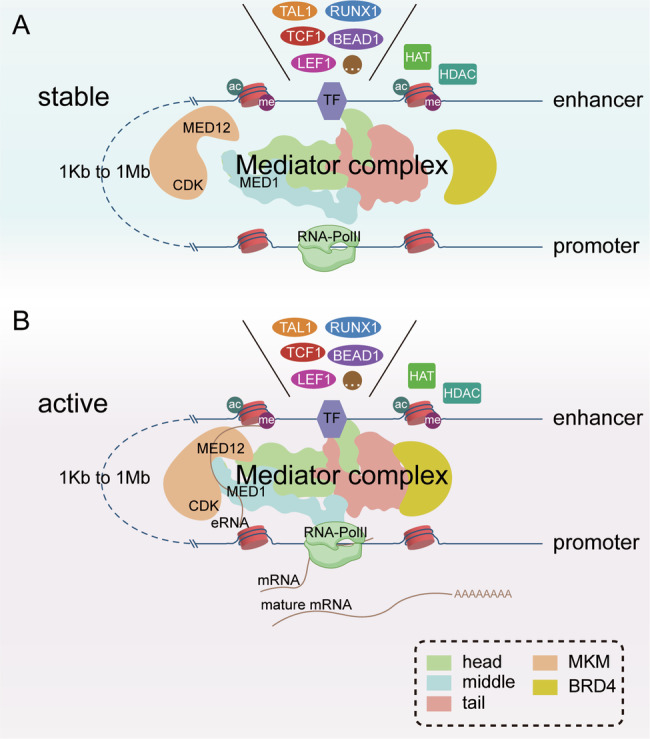
The potential mechanism of enhancer-promoter looping in T cells. The key elements in enhancer-promoter looping include TFs, Mediator complex (including head, middle, and tail, a total of three parts), MKM, BRD4, and RNA-PolII. The acetylation-related key enzymes, HAT and HDAC, are also vital regulated factors. **A** The stable status of enhancer–promoter looping. Elements are in a dissociated state. Sometimes, histone has a low acetylation level. **B** The active status of enhancer-promoter looping. Every element has a physical binding in space. Enhancer recruits TFs and produces eRNA. Mediator complex, MKM, and BRD4 form one big structure. Subunit MED1 and MED12 have a connection with eRNA. Finally, the enhancer drives the related promoter. Abbreviations: MKM mediator kinase module, BRD4 bromodomain-containing protein 4, MED1 mediator complex subunit 1, MED12 mediator complex subunit 12, TF transcription factor, HAT histone acetyltransferase, HDAC histone deacetylase.

Regarding the regulation of enhancer functions, we mainly focus on blocking enhancer activities. Currently, the most common approach is the use of small molecular chemical compounds to interfere with enhancer-promoter looping. BET inhibitors, which interfere with BRD, and CDK inhibitors, which reduce RNA-PolII phosphorylation levels, are widely used in both clinical and pre-clinical research. Depressingly, most BET/CDK interventional strategies lack specificity and easily raise AEs. Prospectively, we believe that the Mediator complex should be paying more attention as another potential drug target. The human Mediator complex comprises 26 subunits, which form a relatively closed spatial structure when the Mediator complex is activated (Fig. 2). Notably, subunits MED1 and MED12 bind to specific eRNAs, which are associated with the activation of nearby promoters. If we can find some drugs that specifically disrupt any subunit of the abnormal genes’ Mediator complex, this will be progress for the diseases with enhancers dysregulation.

Furthermore, by precisely understanding the enhancer landscape and its interaction with functional genes, we may identify therapeutic targets that can restore normal gene expression patterns and inhibit tumor progression. This innovative approach could offer novel avenues for personalized medicine and significantly improve outcomes for patients with T-cell malignancies and other related diseases.
